# Sympathetic activity regulates epithelial proliferation and wound healing via adrenergic receptor α_2A_

**DOI:** 10.1038/s41598-023-45160-w

**Published:** 2023-10-20

**Authors:** Anne S. ten Hove, Shilpashree Mallesh, Konstantina Zafeiropoulou, Janna W. M. de Kleer, Patricia H. P. van Hamersveld, Olaf Welting, Theodorus B. M. Hakvoort, Sven Wehner, Jurgen Seppen, Wouter J. de Jonge

**Affiliations:** 1 Tytgat Institute for Liver and Intestinal Research, Gastroenterology and Hepatology, Amsterdam Gastroenterology Endocrinology Metabolism, Amsterdam UMC, University of Amsterdam, Meibergdreef 69-71, 1105 BK Amsterdam, The Netherlands; 2 Department of General, Visceral-, Thoracic and Vascular Surgery, University Hospital Bonn, Bonn, Germany

**Keywords:** Cell biology, Gastrointestinal diseases, Neuroscience

## Abstract

Innervation of the intestinal mucosa by the sympathetic nervous system is well described but the effects of adrenergic receptor stimulation on the intestinal epithelium remain equivocal. We therefore investigated the effect of sympathetic neuronal activation on intestinal cells in mouse models and organoid cultures, to identify the molecular routes involved. Using publicly available single-cell RNA sequencing datasets we show that the α_2A_ isoform is the most abundant adrenergic receptor in small intestinal epithelial cells. Stimulation of this receptor with norepinephrine or a synthetic specific α_2A_ receptor agonist promotes epithelial proliferation and stem cell function, while reducing differentiation in vivo and in intestinal organoids. In an anastomotic healing mouse model, adrenergic receptor α_2A_ stimulation resulted in improved anastomotic healing, while surgical sympathectomy augmented anastomotic leak. Furthermore, stimulation of this receptor led to profound changes in the microbial composition, likely because of altered epithelial antimicrobial peptide secretion. Thus, we established that adrenergic receptor α_2A_ is the molecular delegate of intestinal epithelial sympathetic activity controlling epithelial proliferation, differentiation, and host defense. Therefore, this receptor could serve as a newly identified molecular target to improve mucosal healing in intestinal inflammation and wounding.

## Introduction

Autonomic neurons are known to influence cells within the intestinal muscularis externa and mucosa through the release of catecholamines like (nor)epinephrine acting on adrenergic receptors (ARs). Such neuro-immune communication is relevant in for instance the way chronic stress is perceived and is demonstrated through the immune-dampening effect of electrical stimulation of the mesenteric nerve plexus in mouse colitis models^1^. Moreover, norepinephrine is a strong immunosuppressive neurotransmitter in vitro^2,3^. Which cells are prone to immune regulation through this route is less well described^4^.

It has been demonstrated that autonomic neuronal activity can also directly affect intestinal epithelial functions such as proliferation and differentiation^5–9^. This is critical in the process of intestinal epithelial turnover and self-renewal maintaining a functional mucosal barrier, which is impaired in wounded conditions such as inflammatory bowel diseases (IBD), and stress- or surgery-induced injury. Sympathetic nerves reach to the level of enteric myofibroblasts and intestinal epithelial cells, and ARs are expressed in these cells^7,9–11^ enabling immune regulation and control of homeostasis through this route. Enteric glial cells have been demonstrated to promote intestinal mucosal healing^12^. The loss of sympathetic innervation in intestinal biopsies of areas with mucosal damage in patients with Crohn’s disease suggests that sympathetic neuronal activity may also be required to achieve mucosal healing^13^. The AR isoform α_2A_ was suggested to play a role in this phenomenon^6,9^. Yet, thus far mechanistic details and in vivo effects have not been entirely characterized^14^.

In this study, we aimed to investigate the role of sympathetic neuronal activity on intestinal cell function and identify responsible receptors and involved pathways. To this end, we reanalyzed publicly available single-cell RNA sequencing (scRNA-seq) datasets to study the expression of ARs in human and mouse intestinal epithelium and assessed the effects of specific AR agonists on mouse small intestinal organoids. In vivo mouse models with specific AR agonist treatment, mesenteric nerve plexus denervation, and intestinal wounding were used to confirm observed effects. Our results show that adrenergic neuronal activity, in particular through α_2A_-AR, is an important factor in intestinal regeneration and could be a newly identified molecular target to improve mucosal healing in intestinal inflammation.

## Materials and methods

### Ethics statement

Human fetal intestinal tissue (gestational age 19–20 weeks) was obtained by the HIS Mouse Facility of the Amsterdam University Medical Center (AUMC) with a written informed consent obtained from all donors for the use of the material for research purposes. The tissue was obtained with approval of the medical ethical committee of the AUMC together with approval of the experimental procedures by the HIS Mouse Facility. All experiments were performed according to guidelines and regulations that are mentioned in the Amsterdam UMC Research Code regulations and in accordance with Dutch law.

### Mice

Female C57BL/6N inbred mice (8–12 weeks old) were purchased from Charles River Laboratories (Maastricht, the Netherlands) and acclimated for 1 week before performing experiments. The animals were housed under specific pathogen-free conditions in the animal facility at the Amsterdam University Medical Centers, location AMC, Amsterdam, the Netherlands. Animals were maintained on a 12/12 light/dark cycle under constant temperature (20 °C ± 2 °C) and humidity (55%) conditions with ad libitum drinking water and chow. Mice were handled according to the guidelines of the Animal Research Ethics Committee of the University of Amsterdam. High flow carbon dioxide was used for euthanasia. Experiments were approved by the Dutch Central Animal Experiments Committee. Individual experiments were revised and approved by the Animal Research Ethics Committee of the University of Amsterdam. Prior to the experiments protocols were approved by this same committee. This study is in accordance with the ARRIVE (Animal Research: Reporting of In Vivo Experiments) guidelines.

### UK 14,304 oral gavage

UK 14,304 (Ref. No. U104-25MG; Sigma-Aldrich) was dissolved in DMSO (Sigma-Aldrich) and further diluted in dH_2_O. UK 14,304 was administered with oral gavage at a concentration of 2.5 mg/kg body weight in a total volume of 100 μL for either 3 or 21 consecutive days. Control animals were given dH_2_O containing 2% DMSO as vehicle for equal durations. During the experiment body weights and behavior were recorded. After 3 and 21 days, mice were euthanized and tissues were collected. Intestinal tissues were cut in half longitudinally for histopathology and RNA isolation.

### Anastomotic healing mouse model

Mice were anesthetized with 2% isoflurane/O_2_ and were given buprenorphine as an analgesic (both intra-operatively and twice post-operatively). A 1 cm midline laparotomy was performed, after which the cecum was exteriorized and the ascending colon was macroscopically transected 1 cm from the cecum without harming the blood supply. Consequently, an end-to-end anastomosis was performed using six separate sutures. Next, the colon was repositioned and the abdomen was closed. During the procedure, the intestines were kept moist.

In the case of mesenteric denervation, sympathetic denervation of the intestine was achieved by transecting the superior mesenteric nerve bundle along the mesenteric artery^15^. This was performed in the same surgical procedure as the creation of the intestinal anastomosis. If mice received UK 14,304, they were first subjected to 3 days of daily oral gavage before surgery. In all experiments using the anastomotic healing mouse model, mice recovered for 5 days before euthanasia.

### Mouse intestinal crypt isolation and organoid culture

Small intestinal organoids were established from female wild-type C57BL/6 mice. Small intestines were opened longitudinally, and villi were scraped using a cover slip. The intestine was cut into small pieces (2–4 mm), transferred to a 50 mL tube, and washed with 10 mL cold PBS by pipetting up and down approximately 10 times. Afterwards, the pieces were incubated in 2 mM 25 mL EDTA (Ref. No. E7889; Sigma-Aldrich) in PBS for 30 min at 4 °C while constantly rotating (100 RPM). The supernatant was removed and 10 mL of ice-cold PBS containing 10% FBS was added and pipetted up and down a few times. The first fraction of the supernatant was discarded, while the 3 subsequent fractions were collected in a 50 mL tube after passing a 70 μM cell strainer. The crypts were pelleted using a centrifuge for 5 min at 800 RPM and collected in Matrigel (Ref. No. BD356231; Corning Life Sciences B.V., Amsterdam, the Netherlands) (20 μL per well; 48-wells plate) for seeding. The plate was left in the incubator (37 °C; 5% CO_2_) for 15–30 min. Afterwards, 250 μL of complete growth medium was added to the well. Typically, the medium was refreshed every 2–3 days and organoids were passaged weekly in a split ratio of 1:3 to 1:5 depending on density^16,17^. Organoids were stimulated for indicated time periods by the addition of indicated agonists (norepinephrine (Ref. No. A7257; Sigma-Aldrich) or UK 14,304 (Ref. No. U104; Sigma-Aldrich)).

### Fetal crypt isolation and monolayer culture

Small intestine was collected from 19 weeks old aborted human fetuses. Tissue was cut longitudinally, washed in cold PBS, cut into small pieces and washed 5 times in cold PBS. Tissue was incubated for 1 h at 4 °C, vigorously shaken in a dissociation mix of Advanced DMEM/F12 (Ref. No. 12634010; Thermo Fisher Scientific) containing 5 mM EDTA, 2 mM dithiotreitol (Ref. No. D0632; Sigma-Aldrich), penicillin/streptomycin, and FBS. Supernatants containing intestinal crypts was collected and centrifuged for 10 min at 950 rpm. Supernatant was removed and cells were washed in 10 mL Advanced DMEM/F12. Cells were centrifuged for 5 min at 950 rpm. Pellet was resuspended in Matrigel in a 24-well cell culture plate. Plate was incubated for 15 min at 37 °C and Intesticult Organoid Growth Medium (OGM, Ref. No. 06010; STEMCELL Technologies, Vancouver, Canada) was added. OGM was supplemented with penicillin/streptomycin, primocin (Ref. No. ant-pm-05; InvivoGen), and Rho kinase inhibitor IV (STEMCELL Technologies). Medium was refreshed every 2–3 days and cells were passaged every 5–8 days. Cells were harvested for monolayer culture after two passages. Single cell suspension was acquired by incubating with 1–2 mL TripLE Express Enzyme (Ref. No. 12605010; Thermo Fisher Scientific) per donor at 37 °C and vigorously pipetting.

### Immunofluorescence

Intestinal samples were fixated in 4% paraformaldehyde (PFA; Ref. No. 1.04005.1000, VWR International B.V., Amsterdam, the Netherlands). Afterwards, tissues were processed with a tissue processor (Shandon Excelsior ES, Thermo Fisher Scientific). Finally, dehydrated samples were paraffin-embedded in the embedding station (HistoCore Arcadia, Leica Microsystems B.V., Amsterdam, the Netherlands), cut in 5 μm-thick sections on a microtome (Microm HM 340E, Thermo Fisher Scientific), and dried overnight at 37 °C. Slides were deparaffinized with xylene and rehydrated in a graded series of ethanol. For antigen retrieval, tissues were heated in 0.01 M sodium citrate solution (pH 6.0) at 95 °C for 20 min. Non-specific binding was prevented by incubation with PBT (PBS, 1% BSA, 1% Triton-X). Tissue sections were incubated overnight with the primary antibody at 4 °C. Incubation with the secondary antibody was performed at room temperature for 1 h. The following antibodies were used: Lysozyme (1:500; Ref. No. A0099, Agilent, Santa Clara, CA, USA), Muc2 (1:200; Ref. No SC-15334, Santa Cruz Biotechnology, Dallas, TX, USA). ProLong Antifade Mountant with DAPI was used for mounting and staining nuclei. Invitrogen, Images were captured using an Olympus BX51 microscope.

For organoid samples, organoids were first treated with Cell Recovery Solution (Ref. No. 354253, Corning Life Sciences B.V.), fixed with 4% PFA for 30 min, and embedded in HistoGel (Ref. No. HG-4000-012, Thermo Fisher Scientific, Bleiswijk, the Netherlands).

### RNAscope (in situ hybridization)

RNAscope in situ hybridization (Advanced Cell Diagnostics, Biotechne, Newark, NJ, USA) was performed according to the manufacturer’s protocol Formalin-Fixed Paraffin-Embedded Sample Preparation and Pretreatment for RNAscope 2.5 assay and RNAscope 2.5 HD Detection Reagent-RED. In brief, slides were deparaffinized, and treated with hydrogen peroxide, target retrieval reagents, and protease. Afterwards, samples were treated with hybridizations with target-specific probes and amplifiers. All components were provided by the manufacturer (RNAscope 2.5 HD Reagent Kit-RED; Ref. No. 322350). The following probes were used: Hs-ADRA2A (Ref. No. 602791), and Mm-Adra2a (Ref. No. 425341). After probes were detected by Fast RED, slides were washed 3 times with PBS, followed by the staining with the primary antibody against Lysozyme overnight at 4 °C. After washing with PBS, sections were stained with a secondary antibody (Alexa Fluor 488 in 1:200; Ref. No. A11008, Invitrogen, Thermo Fisher Scientific), at room temperature for 60 min. Slides were washed in PBS and mounted with ProLong Gold Antifade reagent containing DAPI. Pictures were taken with a Leica DM6000 microscope using LAS AF software. Image processing was done with ImageJ software (v1.50i)^18^.

### IncuCyte

Following the seeding of the organoids, the 48-wells plates were placed in the IncuCyte (Essen BioScience, Newark, UK) for 5 days. Images were acquired automatically in phase contrast every 2 h at 4x. After completion, pictures were analyzed by measuring the circumferences of 11 organoids throughout the 5 days.

### 5-ethynyl-2′-deoxyuridine proliferation assay

Organoids were seeded on 48-wells plates and stimulated with agonists for 3 days. ClickIT™ EdU kit Plus EdU Alexa Fluor 647 Imaging Kit (Ref. No. C10640, Thermo Fisher Scientific) was used according to the manufacturer’s instructions. Briefly, organoids were cultured with 0,2% 5-ethynyl-2′-deoxyuridine (EdU) diluted in ENR for 16 h to allow incorporation of fluorescently labeled EdU into newly synthesized DNA marking proliferating cells. Afterwards, cells were harvested with Cell Recovery Solution (Corning) for 30 min on ice at 4 °C, fixed with 4% PFA, permeabilized, and labeled with ClickIT-647. Afterwards, FACS was performed using a BD LSRFortessa (BD Biosciences, Vianen, the Netherlands) and analysis was done with FlowJo (v10.6.1, BD Biosciences).

### Apoptosis assay

Organoids were seeded on 48-wells plates and stimulated with agonists for 3 days. Afterwards, organoids were harvested with Cell Recovery Solution and a single-cell suspension was made by use of incubation with TrypLE for 10 min at 37 °C. Cells were subsequently washed with 10% FCS/PBS and processed with FITC Annexin V Apoptosis Detection Kit II (Ref. No. 556570; BD Biosciences). Staining was assessed by FACS followed by data analysis using FlowJo software.

### ECIS wound healing assay

8 well Electric Cell-Substrate Impedance Sensing (ECIS) array (8W10E) was coated with 10 mM L-cysteine, followed by Rat Collagen Type I (Ref. No. 50201; Ibidi GmbH, Gräfelfing, Germany) coating of 8 ug per well. Electrodes were stabilized with the electrical stabilization setting of the ECIS. Baseline was measured for 30 min. A single cell suspension of human fetal organoids (gestational age 19 weeks) was seeded at a density of 250.000 cells/well in 400 uL OGM. Cells were incubated until impedance stabilized, indicating a confluent monolayer. Wounding was performed for 60 s with a frequency of 50,000 Hz and current of 5500 μA. Cells were refreshed every 2–3 days.

### RNA isolation, cDNA synthesis, quantitative PCR analysis, and RNA sequencing

Total RNA was isolated from samples using Bioline ISOLATE II RNA Mini Kit (GC Biotech, Alphen aan den Rijn, the Netherlands) according to the manufacturer’s protocol. cDNA was synthesized by use of deoxynucleotide triphosphates (Thermo Fisher Scientific), Random primers (Promega, Leiden, The Netherlands), Oligo dT primers (Sigma, Zwijndrecht, The Netherlands), Revertaid, and Ribolock (both Thermo Fisher Scientific). Quantitative PCR (qPCR) was performed with SensiFAST SYBR No-ROX (GC Biotech) and a LightCycler 480 II (Roche). Analysis was performed with LinRegPCR software. For normalization reference genes were used that were selected with GeNorm software. Primers (all from Sigma) are listed in Supplementary Table 1.

For RNA sequencing, RNA quality was assured using the TapeStation 4200 (Agilent). Samples with an RNA integrity number (RNA) > 8 were used for further analyses. mRNA was converted into cDNA with the KAPA mRNA HyperPrep Kit (Roche). cDNA preparation for sequencing was done using the HiSeq4000 at the Core Facility Genomics, Amsterdam UMC, in a 50 bp single-ended fashion with a depth of 40 M reads per sample. Raw reads were checked for quality with FastQC (v0.11.8) and MultiQC (v1.7)^19,20^. Mapping was done with STAR (v2.7.3) against mouse genome GRCm38^21^. Post-alignment processing was done using SAMtools (v1.9), whereupon reads were counted using featureCounts in the Subread package (v.1.6.4)^22,23^. Gene features were obtained from Ensembl (v98)^24^. Subsequently, counts were imported and analyzed using Rstudio with packages obtained from Bioconductor (v3.10)^25^. DESeq2 (v1.30.0) was used to perform differential expression analyses, and apeglm was used for shrinkage^26^. Pheatmap (v1.0.12) and ggplot2 (v3.3.3) were used to create plots^27–29^.

### Microbiota profiling

Murine fecal pellets were processed using the PSP Spin Stool DNA Plus Kit (Ref. No. 1038110300, STRATEC Molecular GmbH, Germany) according to the manufacturer’s protocol. In brief, DNA was extracted from 1 or 2 murine fecal pellets depending on their size using mechanical lysis and the PSP Spin Stool DNA Plus Kit. After, the samples were placed in Lysing Matrix E tubes with 1,400 μl stabilizer. Mechanical lysis was accomplished using the FastPrep in 3 repetitive rounds of 30 s at 6.5 m/s and subsequent cooling of 30 s on ice. Further extraction was done by heating at 97 °C for 15 min, subsequent cooling on ice for 1 min and centrifugation at 13,400 g for 1 min. Supernatant was transferred to the PSP InviAdsorb. Negative controls (DNA-free water) were processed equally. The Nanodrop 1000 spectrophotometer (Thermo Fisher Scientific) was used to measure DNA concentrations.

Using a single-step PCR protocol targeting the V3–V4 region 16S rRNA gene amplicons were generated^30^. The PCR reaction in 20 μl was performed with the following thermocycling conditions: denaturation at 98 °C, 25 cycles of denaturation (98 °C for 10 s), annealing (55 °C for 20 s), extension (72 °C for 90 s), and final extension at 72 °C for 10 min. PCR products were purified using Ampure XP beads (Ref. No. A63882, Beckman Coulter Life Sciences, Woerden, The Netherlands), and purified products were pooled in an equimolar manner. Libraries were sequenced with a MiSeq platform using V3 chemistry with 2 × 251 cycles.

Forward and reverse reads were truncated to 240 and 210 bases, respectively, and merged using USEARCH. Merged reads not passing the Illumina chastity filter had an expected error rate higher than 2 or were shorter than 380 bases. Amplified sequence variants (ASVs) were inferred for each sample individually with a minimal abundance of 4 reads^31^. Unfiltered reads were mapped against the collective ASV set to determine the abundances. Taxonomy was assigned using SILVA 16S ribosomal database V132^32^.

### Statistical analysis microbiota profiling

Microbial multivariate statistical analysis was performed in R statistical environment (version 4.0.4) using the packages phyloseq (version 1.34.0)^33^ and vegan (version 2.5.7). Samples were rarefied to 50,000 reads. Alpha diversity indices were calculated (i.e., species richness and Shannon entropy). Microbial composition was assessed using non-metric multidimensional scaling plots (NMDS) on weighted UniFrac distance at ASV level based. The latter uses species abundance information and weights the branch length with abundance difference. Non-parametric permutational multivariate analysis of variance (PERMANOVA) was applied using the vegan Adonis function on the weighted UniFrac distance matrix. Differences in genus relative abundances between groups were found using the DESeq2 method (version 1.30.1)^27^. Basemean, log2-fold change, and non-adjusted and adjusted *P*-values of the aforementioned analyses at ASV and genus levels are reported in Supplementary Tables 2 and 3.

### Single-cell RNA-sequencing database studies

A publicly available single-cell RNA-sequencing dataset of mouse small intestine (GSE92332) was downloaded from the Gene Expression Omnibus (GEO) and imported into Rstudio (v1.3.1093)^34,35^. Seurat (v3.1.5) was used to import, integrate, and cluster the data^36^. Plots were made using ggplot2 (v3.3.3)^29^.

In the GSE92332 dataset, cell filtering on dead cells was already performed by the original authors. Cells were filtered based on *Epcam* expression. Louvain clustering analysis identified 12 clusters, which were annotated using known markers: stem cells (*Lgr5*, *Ascl2*, *Slc12a2*, *Axin2*, *Olfm4*, *Gkn3*), enterocytes (*Alpi*, *Apoa1*, *Apoa4*, *Fabp1*), Paneth cells (*Lyz1*, *Defa17*, *Defa22*, *Defa24*, *Ang4*), goblet cells (*Muc2*, *Clca3*, *Tff3*, *Agr2*), enteroendocrine cells (*Chga*, *Chgb*, *Tac1*, *Tph1*, *Neurog3*), and tuft cells (*Dclk1*, *Trpm5*, *Gfi1b*, *Il25*)^35^. Subsequently, the expression of adrenergic receptors was assessed.

For analysis of human samples, publicly available single-cell RNA-sequencing datasets from the human small intestine, colon, and rectum (GSE125970) were downloaded from the Sequence Read Archive (SRA)^37^. Alignment was done against GRCh38 using Cell Ranger software (v3.1.0; 10 × Genomics, Inc., Pleasanton, CA, USA). All cells were first filtered for dead cells, identified by a low (< 200) or high (> 4000) gene count. Next, epithelial cells were selected based on *EPCAM* expression. Clustering analysis identified 31, 26, and 22 clusters for ileal, colonic, and rectal samples, respectively. Specifically, we identified stem cells (*LGR5*, *RGMB*, *SMOC2*, *ASCL2*), transit amplifying cells (*MKI67*, *PCNA*, *TOP2A*, *CCNA2*, *MCM5*), progenitor cells (*SOX9*, *CDK6*, *MUC4*, *FABP5*, *PLA2G2A*, *LCN2*), enterocytes (*ALPI*, *SLC26A3*, *TMEM37*, *FABP2*), Paneth cells (*LYZ*, *CA7*, *SPIB*, *CA4*, *FKBP1A*), goblet cells (*ZG16*, *CLCA1*, *FFAR4*, *TFF3*, *SPINK4*), enteroendocrine cells (*CHGA*, *CHGB*, *CPE*, *NEUROD1*, *PYY*), and tuft cells (*POU2F3*, *GFI1B*, *TRPM5*).

### Data presentation and statistical analysis

Graphs and statistical analyses were made using Prism 8.3.0 (GraphPad Software, La Jolla, CA, USA). Data are shown as mean if distributed normally, or as median if not distributed normally, and/or individual data points. A *P* < 0.05 was considered significant. Data were compared with the independent T-test or Mann–Whitney U test as appropriate. One-way ANOVA and Dunnett’s multiple comparison tests were used for multiple groups.

## Results

### Adrenergic receptor isoform α_2A_ is expressed in most intestinal epithelial cell types

**Figure 1:**
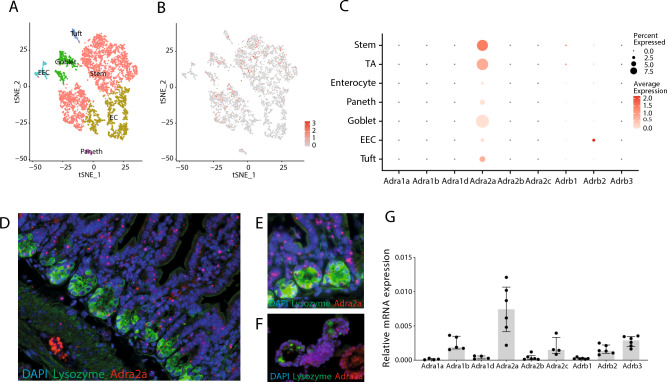
Intestinal epithelial cell expression of adrenergic receptors. (**A**) Single-cell RNA-sequencing of mouse small intestinal epithelial cells. Data were obtained from GSE92332^35^. t-distributed stochastic neighbor embedding (t-SNE) visualization of the unsupervised clustering analysis of all cells. (**B**) Visualization of the expression of *Adra2a* with t-SNE. (**C**) Dot Plot visualization of the expression of adrenergic receptors in cell type classes. (**D**,**E**) In situ hybridization (RNAscope) for *Adra2a* mRNA in mouse small intestine in combination with immunofluorescent stainings with DAPI for nuclei (blue) and lysozyme (green). Single dots (red) represent expression of a single *Adra2a* mRNA copy. Magnification: 20x (D) or 40x (E). (**F**) In situ hybridization for *Adra2a* with immunofluorescent stainings for nuclei and lysozyme in mouse small intestinal organoids. (**G**) Quantitative RT-PCR analysis for adrenergic expression in mouse small intestinal organoids (*n* = 6). Data are shown as median with interquartile range and individual data points.

We sought to establish a detailed insight in the response of the intestinal epithelium to sympathetic neuronal activation, as this is currently not well described^38^. First, we investigated the expression of ARs in intestinal epithelium on a single-cell level. To this end, we reanalyzed a murine single-cell RNA sequencing (scRNA-seq) dataset^35^. Cell clusters were identified in t-distributed stochastic neighbor embedding (t-SNE) maps using known marker genes (Fig. 1A)^35^. Interestingly, α_2A_-AR was the only receptor of the ones we investigated that was found to be expressed in the small intestinal epithelium, and prominent expression was shown throughout various epithelial cell types, such as stem cells, Paneth cells, goblet cells, and enteroendocrine cells (Fig. 1B,C). For human AR expression profiles, the human scRNA-seq dataset of Wang (GSE125970)^37^ was studied, showing similar results with α_2A_-AR being expressed most profoundly throughout the ileum, colon, and rectum. Yet, although expression of this specific AR was chiefly in stem cells in mice, in humans this was in Paneth cells (Supplementary Fig. 1). As immunohistochemistry for ARs is difficult because of the lack of specificity of available antibodies, we aimed to validate our findings by use of in situ hybridization with RNAscope. In line with our scRNA-seq data and earlier research^7^, expression of α_2A_-AR was found throughout the whole intestinal epithelial layer, both in the villus and stem cell compartment (Fig. 1D,E, Supplementary Fig. 2A). Similar expression patterns were found in mouse small intestinal organoids as demonstrated by RNAscope (Fig. 1F, Supplementary Fig. 2B) and qPCR (Fig. 1G). These data suggest that intestinal epithelial cells are susceptible for AR stimulation through α_2A_-AR.

### General and specific α_2A_-AR stimulation results in increased epithelial cell proliferation

**Figure 2:**
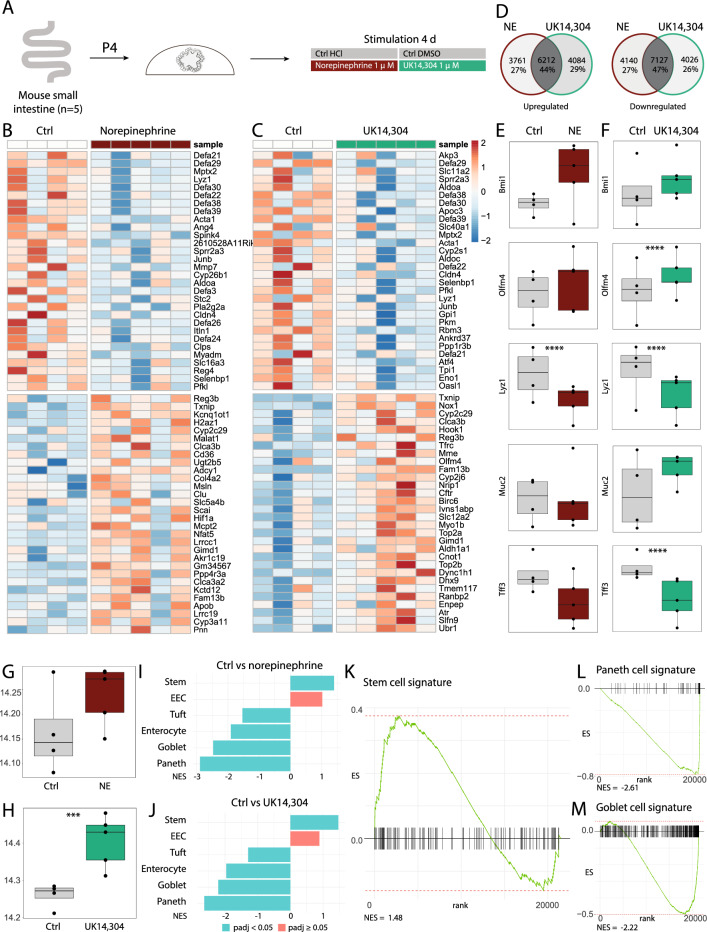
Bulk RNA-sequencing analysis of organoids stimulated with norepinephrine or UK 14,304. (**A**) Experimental set-up. Mouse small intestinal organoids were grown and after four passages they were stimulated with either norepinephrine or UK 14,304 1 μM (or vehicle controls) for four days. Afterwards they were harvested and processed (*n* = 5). (**B**,**C**) Heat map visualizing the top 30 upregulated and downregulated genes upon stimulation with norepinephrine (**B**) or UK 14,304 (**C**) and vehicle control. (**D**) Venn diagrams showing overlap of upregulated and downregulated genes upon stimulation with norepinephrine or UK 14,304. (**E**) Single gene expression analysis of intestinal cell markers *Bmi1* (*P* = 0.089), *Olfm4* (*P* = 0.116), *Lyz1* (*P* < 0.0001), *Muc2* (*P* = 0.927), and *Tff3* (*P* = 0.160) in norepinephrine versus vehicle control treated organoids. (**F**) Single gene expression analysis of intestinal cell markers *Bmi1* (*P* = 0.217), *Olfm4* (*P* < 0.0001), *Lyz1* (*P* < 0.0001), *Muc2* (*P* = 0.089), and *Tff3* (*P* < 0.0001) in UK 14,304 versus vehicle control treated organoids. (**G**,**H**) Single gene expression analysis of cell proliferation marker *Mki67* in norepinephrine (G; *P* = 0.100) or UK 14,304 (H; *P* = 0.0001) versus vehicle control treated organoids. (**I**,**J**) Gene Set Enrichment Analysis (GSEA) of cell type signatures in norepinephrine (**I**) or UK 14,304 (**J**) treated organoids versus vehicle controls. Blue bars indicate adjusted *P* < 0.05, and red bars indicate adjusted *P* > 0.05. (**K**) GSEA of signature of stem cells in UK 14,304 treated organoids versus vehicle controls (Normalized enrichment score (NES) = 1.48; *P* = 0.006). (**L**) GSEA of signature of Paneth cells in UK 14,304 treated organoids versus vehicle controls (NES = − 2.61; *P* = 0.002). (**M**) GSEA of signature of goblet cells in UK 14,304 treated organoids versus vehicle controls (NES = − 2.22; *P* = 0.003). Statistical significance is indicated as follows: *P < 0.05, **P < 0.01, ***P < 0.001, ****P < 0.0001. Data are shown in boxplots.

To explore the pathways that are activated upon intestinal epithelial AR stimulation, mouse organoids were stimulated with the general α- and β-AR agonist norepinephrine and the complete potent and specific α_2_-AR agonist UK 14,304 (5-bromo-6-[2-imidazolin-2-ylamino]-quinoxaline) using concentrations based on literature (Fig. 2A)^6,39^. The top 30 upregulated and downregulated genes that were differentially expressed between vehicle control treated organoids and norepinephrine or UK 14,304 treated organoids are shown in heat maps (Fig. 2B,C). The Venn diagrams illustrate that upregulated and downregulated genes are greatly overlapping between norepinephrine and UK 14,304 treated organoids (Fig. 2D). To study expression patterns more in detail, expression levels of specific genes encoding stem cell, proliferation, and differentiation genes were assessed. Stem cell marker Olfm4 (*P* < 0.0001) was upregulated upon stimulation, whereas markers of differentiated cell types like Lyz1, and Tff3 (both *P* < 0.0001) were downregulated, in particular in UK 14,304 treated organoids (Fig. 2E,F). Similar changes could be observed in the heat maps with noted downregulation of antimicrobial peptides (defensins, Lyz1) and upregulation of stem cell or proliferation markers (Slc12a2, Top2a). To assess effects on cell proliferation, *Mki67* (encoding for proliferation marker protein Ki67) expression was investigated. Results indicated a marked upregulation of this cell proliferation marker in UK 14,304 treated organoids (*P* = 0.0001), but not in norepinephrine treated organoids (Fig. 2G,H). Since these data demonstrated a distinct difference between stem cell and differentiated cell type markers, we hypothesized that epithelial cell type composition patterns would be altered. To test this, cell-type signatures composed of key transcription factors and specific G-protein coupled receptors were studied. By use of previously identified cell type signatures for Gene Set Enrichment Analysis (GSEA)^35^, we show that the stem cell signature is indeed significantly upregulated, and signatures of tuft cells, enterocytes, goblet cells, and Paneth cells are significantly downregulated, both upon norepinephrine treatment and UK 14,304 treatment when compared to vehicle controls (Fig. 2I,J). Specific cell type signatures upon stimulation with UK 14,304 of stem cells (NES = 1.48, *P* = 0.006), Paneth cells (NES = − 2.61, *P* = 0.002), and goblet cells (NES = − 2.22, *P* = 0.002) are shown in respectively Fig. 2K–M.

**Figure 3:**
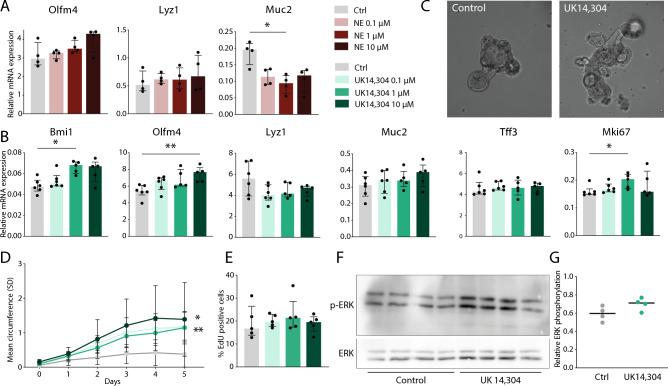
In vitro experiments of organoids treated with norepinephrine or UK 14,304. (**A**) Quantitative RT-PCR of intestinal epithelial markers in mouse small intestinal organoids that were stimulated with norepinephrine in concentrations 0.1 μM, 1 μM, and 10 μM, or vehicle control (*n* = 4). Data are shown as individual data points with median and interquartile range. *Muc2* vehicle control versus norepinephrine 1 μM adj *P* = 0.024. (**B**) Quantitative RT-PCR of intestinal epithelial markers in mouse small intestinal organoids that were stimulated with UK 14,304 in concentrations 0.1 μM, 1 μM, and 10 μM, or vehicle control (n = 6). Data are shown as individual data points with median and interquartile range. *Bmi1* vehicle control versus UK 14,304 1 μM adj *P* = 0.013; *Olfm4* vehicle control versus UK 14,304 10 μM adj *P* = 0.008; *Mki67* vehicle control versus UK 14,304 1 μM adj *P* = 0.033. (**C**) Representative pictures of vehicle treated organoids (left) or UK 14,304 treated organoids (right). (**D**) Visualization of organoid growth upon stimulation with UK 14,304 measured by circumference using the IncuCyte. Data are shown as median with standard deviation (SD) (*n* = 11). Statistical significant differences were assessed at day 5 using: vehicle control versus UK 14,304 0.1 μM (adj *P* = 0.003), 1 μM (adj *P* = 0.003), and 10 μM (adj *P* = 0.013). (**E**) FACS analysis of cell proliferation marker 5-ethynyl-2′-deoxyuridine (EdU) in UK 14,304 treated organoids versus vehicle control (*n* = 5). Data are shown as individual data points with median and interquartile range. (**F**) Western blot analysis of p-ERK1/2 and ERK1/2 in lysates of organoids stimulated with UK 14,304 or vehicle control (*n* = 4). (**G**) Quantification of western blot results. Data are shown as individual data points with median. *P* = 0.091, unpaired t test. Statistical significance is indicated as follows: *P < 0.05, **P < 0.01, ***P < 0.001.

We next validated our earlier RNA-seq results by qPCR showing increased expression levels of stem cell markers and decreased expression of differentiated cell type markers, although not all significant (Fig. 3A,B). Furthermore, expression of *Mki67* was upregulated upon treatment with UK 14,304 (adj *P* = 0.033), in line with earlier data. Therefore, we postulated that increased proliferation upon AR stimulation would lead to increased growth and thus increased organoid sizes. Thereupon, circumference measurements to assess organoid size were performed for five consecutive days on multiple organoids stimulated with the same agonist whilst in culture in the IncuCyte (representative pictures shown in Fig. 3C). Indeed, organoids presented increased growth in all three concentrations compared to vehicle-treated organoids (vehicle control vs. UK 14,304 0.1 μM (adj *P* = 0.003), 1 μM (adj *P* = 0.003), and 10 μM (adj *P* = 0.013) (Fig. 3D). Successively, EdU labeling for sixteen hours was performed in organoids to mark proliferating cells, but increase of proliferation could not be shown through this method (Fig. 3E). To confirm that increased organoid size in the vehicle stimulated group was not the effect of increased apoptosis (leading to extrusion of dead cells into the lumen and hence growth of organoids), an apoptosis assay was performed using combined propidium iodide/Annexin V stainings to detect early apoptosis and general cell death. Levels were similar in stimulated versus vehicle-treated samples, implying apoptosis was not causative for these changes (Supplementary Fig. 3). It was previously demonstrated that altered MAPK signaling caused increased epithelial cell proliferation^6^. To support this, we performed a western blot on lysates of stimulation organoids with either UK 14,304 or vehicle control. In consistence with earlier studies, an increase in phosphorylated Erk could be noted upon UK 14,304 treatment when compared to vehicle control (Fig. 3F,G). This is in line with earlier research showing enhanced ERK phosphorylation by α_2A_-AR activation through both a Src-dependent and a Src-independent pathway^40^. Altogether, these data suggest that AR stimulation leads to increased cell proliferation, likely due to the promotion of stem cell functions through α_2A_-AR. The consequence of this stimulation is an altered epithelial cell differentiation.

### α_2A_-AR stimulation results in increased epithelial cell proliferation in vivo

**Figure 4:**
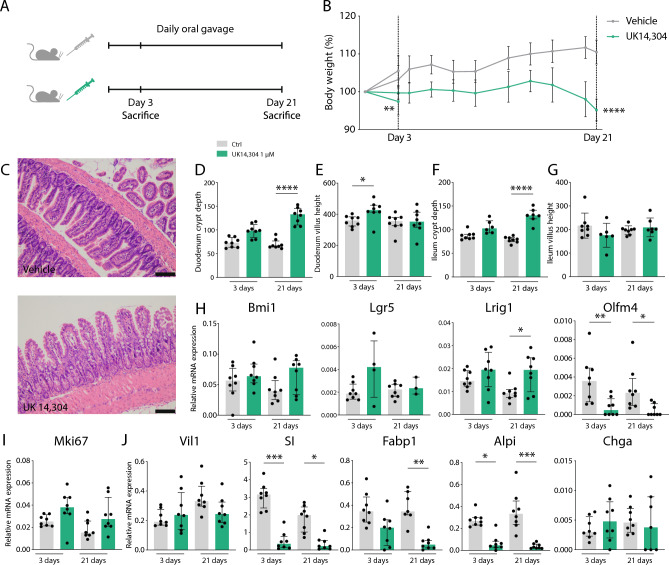
Assessment of α_2A_-AR stimulation on stem cell function in vivo. (**A**) Experimental set-up. Wild-type mice were treated with UK 14,304 or vehicle control via daily oral gavage for 3 or 21 days (*n* = 8). (**B**) Visualization of body weight changes normalized for weight at start of experiment. Results are shown as mean with standard deviation (SD) (*P* = 0.010 at 3 days, *P* < 0.0001 at 21 days). (**C**) Representative pictures of H&E stains of ileum samples of mice treated with vehicle control or UK 14,304 for 21 days. (**D**–**G**) Quantification of crypt depth or villus height measurements. Data are shown as individual data points with median and interquartile range (*n* presents the mean of 25 measurements of 8 individual mice). (**D**) Duodenum crypt depth (vehicle control vs. UK 14,304 (21 days) *P* < 0.0001). (**E**) Duodenum villus height (vehicle control vs. UK 14,304 (3 days) *P* = 0.038). (**F**) Ileum crypt depth (vehicle control vs. UK 14,304 (21 days) *P* < 0.0001). (**G**) Ileum villus height (ns). (**H**) Quantitative RT-PCR (qPCR) analysis of expression of epithelial stem cell markers *Bmi1*, *Lgr5*, *Lrig1* (vehicle control vs. UK 14,304 21 days *P* = 0.035), and *Olfm4* (vehicle control vs. UK 14,304 3 days *P* = 0.009; 21 days *P* = 0.021) in ileum. Data are shown as individual data points with median and interquartile range. (**I**). qPCR analysis of proliferation marker *Mki67* expression in ileum. Data are shown as individual data points with median and interquartile range. (**J**). qPCR analysis of expression of differentiated cell type markers *Vil1*, *SI* (vehicle control vs. UK 14,304 3 days *P* = 0.0002; 21 days *P* = 0.015), *Fabp1* (vehicle control vs. UK 14,304 21 days *P* = 0.001), *Alpi* (vehicle control vs. UK 14,304 3 days *P* = 0.015; 21 days *P* = 0.0002), and *Chga* in ileum. Data are shown as individual data points with median and interquartile range. Statistical significance is indicated as follows: *P < 0.05, **P < 0.01, ***P < 0.001, ****P < 0.0001.

To confirm the effects in an in vivo model, we administered the same compound, UK 14,304, to healthy wild-type mice via daily oral gavage. To study both acute and chronic effects, this was done for either 3 or 21 days (Fig. 4A). Treatment led to more active behavior of mice, and looser fecal pellets compared to control mice, accompanied by body weight changes (*P* < 0.0001, Fig. 4B). Upon euthanasia, intestinal tissues were harvested and assessed for mRNA levels and histology. H&E stainings were assessed for villus length and crypt depth being readouts for epithelial differentiation and proliferation, respectively, in both duodenum and ileum. Representative pictures are shown for ileum samples upon 21 days of vehicle treatment and UK 14,304 treatment (Fig. 4C). Remarkable crypt depth increases were found in both duodenum and ileum samples after 21 days (*P* < 0.0001) (Fig. 4D,F). Villus height was found to be increased upon UK 14,304 oral gavage after 3 days (*P* = 0.038), but this effect disappeared after treatment for 21 days (Fig. 4E). No changes were seen in villus height in ileal samples (Fig. 4G). qPCR analysis was performed to assess expression level change of stem cell and proliferation markers. In line with in vitro data, intestinal stem cell marker *Lrig1* was significantly upregulated after 21 days of oral gavage with UK 14,304 compared to oral gavage with the vehicle control (*P* = 0.035) (Fig. 4H). Other markers like *Bmi1* and *Lgr5* were not significantly upregulated. Unexpected was the downregulation of stem cell marker *Olfm4* (*P* = 0.009). Yet, this is explicable as the glycoprotein Olfactomedin-4 has an acknowledged versatile character since it is both a marker of stem cells and an important mediator of mucosal host defense^41–43^. *Mki67* expression changes were not significant (Fig. 4I). Amongst markers of differentiated cell types *SI* (3 days *P* = 0.0002; 21 days *P* = 0.015), *Fabp1* (*P* = 0.001), and *Alpi* were downregulated (3 days *P* = 0.015; 21 days *P* = 0.0002) (Fig. 4J). Although results were most pronounced in ileum samples, similar effects were seen in duodenum and colon (Supplementary Fig. 4A,B). This experiment confirmed that α_2_-AR specific stimulation leads to increased stem cell function and a decrease of differentiation.

### α_2A_-AR stimulation reduces epithelial antimicrobial peptide expression and host defense

**Figure 5:**
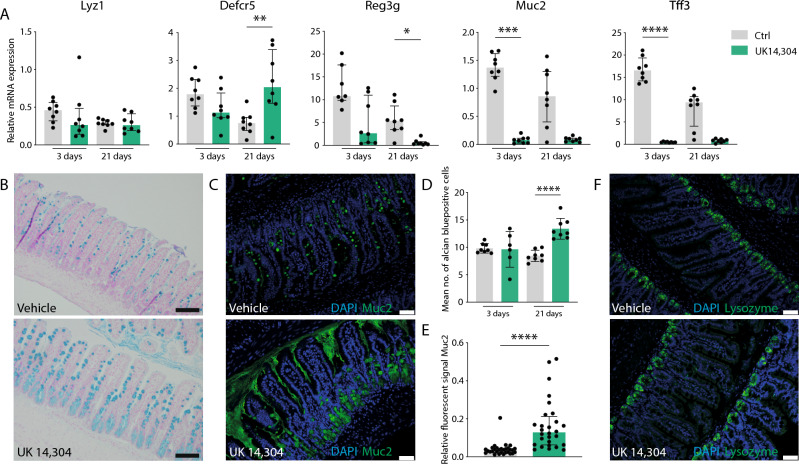
Assessment of α_2A_-AR stimulation on host defense. (**A**) Quantitative RT-PCR (qPCR) analysis of expression of antimicrobial peptides *Lyz1*, *Defcr5* (vehicle control vs. UK 14,304 21 days *P* = 0.003), *Reg3g* (vehicle control vs. UK 14,304 21 days *P* = 0.027), *Muc2* (vehicle control vs. UK 14,304 3 days *P* = 0.0002), and *Tff3* (vehicle control vs. UK 14,304 3 days *P* < 0.0001) in ileum of mice treated with UK 14,304 or vehicle control via daily oral gavage for 3 or 21 days (*n* = 8). Data are shown as individual data points with median and interquartile range. (**B**) Representative pictures of alcian blue stainings of ileum samples of mice treated with vehicle or UK 14,304. (**C**) Representative pictures of immunofluorescent stainings for nuclei DAPI; blue) or Muc2 (green) of ileum samples of mice treated with vehicle or UK 14,304. (**D**) Quantification of (**B**). Data are shown as individual data points with median and interquartile range (*n* presents the mean number alcian blue positive cells of > 15 pictures of 8 individual mice). Vehicle control versus UK 14,304 21 days *P* < 0.0001. (**E**) Quantification of (**C**) showing fluorescent signal of Muc2 relative to DAPI. Data are shown as individual data points with median and interquartile range (*n* presents the mean number alcian blue positive cells of > 15 pictures of 8 individual mice). Vehicle control versus UK 14,304 21 days *P* < 0.0001. (**F**) Representative pictures of immunofluorescent stainings for nuclei (blue) and lysozyme (green). Statistical significance is indicated as follows: *P < 0.05, **P < 0.01, ***P < 0.001, ****P < 0.0001.

We reasoned that the changes in epithelial cell type composition would impact epithelial host defense. To study this, we analyzed the mRNA expression of various secretory peptides like antimicrobial peptides by qPCR analysis. Intriguingly, expression levels of *Defcr5* (*P* = 0.003), *Reg3g* (*P* = 0.027), *Muc2* (*P* = 0.0002), and *Tff3* (*P* < 0.0001) were downregulated immensely upon UK 14,304 treatment, the latter two even already after 3 days of treatment (Fig. 5A). *Lyz1* mRNA expression was unaffected. In agreement with previous reports, contrary to mRNA levels, protein levels of Muc2 increased as assessed by alcian blue stainings and fluorescent stainings (both *P* < 0.0001) (Fig. 5B–E, Supplementary Fig. 2C,D)^44^. This Muc2 increase also evidently leads to morphological changes with goblet cell enlargement in particular (Fig. 4C,D). In line with *Lyz1* qPCR results in Fig. 5A, protein levels of Lysozyme were unchanged upon UK 14,304 treatment (Fig. 5F).

### α_2A_-AR stimulation induces profound changes in the intestinal microbiome

**Figure 6:**
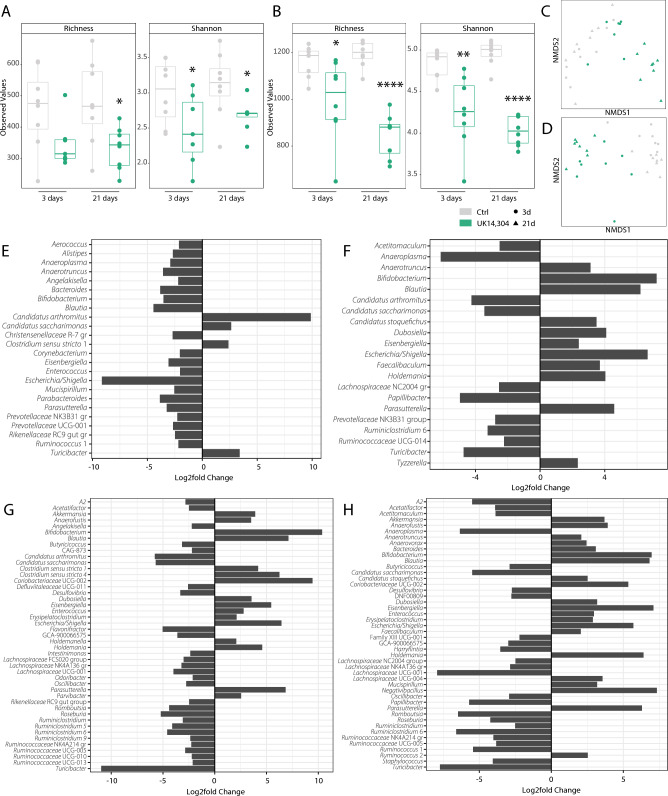
Microbiome analysis upon α_2A_-AR stimulation. (**A**–**H**) Microbial analyses for fecal samples of mice treated with UK 14,304 or vehicle control for 3 or 21 days. (**A**) α-diversity indices including Richness (vehicle control vs. UK 14,304 21 days *P* = 0.014) and Shannon vehicle control versus UK 14,304 3 days *P* = 0.046 and 21 days *P* = 0.03diversity for ileal feces. (**B**) α-diversity indices including Richness (vehicle control vs. UK 14,304 3 days *P* = 0.022 and 21 days *P* < 0.0001) and Shannon (vehicle control vs. UK 14,304 3 days *P* = 0.004 and 21 days *P* < 0.0001) diversity for colonic feces. (**C**) β-diversity for ileal feces (R^2^ = 0.59; *P* = 0.001). (**D**) β-diversity for colonic feces (R^2^ = 0.41; *P* = 0.003). E–H. DESeq2 analysis feces on genus level. (**E**) and (**G**) demonstrate outcomes in ileal samples for 3, and 21 days, respectively, and (**F**) and (**H**) for colonic samples for 3, and 21 days, respectively. Only genera with Log2foldchange < − 2 or > 2 are depicted. Statistical significance is indicated as follows: *P < 0.05, **P < 0.01, ***P < 0.001, ****P < 0.0001.

We were curious to study how these changes in secretory antimicrobial peptides would alter the microbial composition. Hence, 16S bacterial sequencing was performed on ileal and colonic fecal samples of mice treated with UK 14,304 or vehicle control via oral gavage. Significant differences were observed in α-diversity metrics, both in ileal (Richness: vehicle control vs. UK 14,304 21 days *P* = 0.014; Shannon: vehicle control vs. UK 14,304 3 days *P* = 0.046 and 21 days *P* = 0.03) and colonic (Richness: vehicle control vs. UK 14,304 3 days *P* = 0.022 and 21 days *P* < 0.0001; Shannon: vehicle control vs. UK 14,304 3 days *P* = 0.004 and 21 days *P* < 0.0001) feces (Fig. 6A,B), particularly in 21 days treated groups. β-diversity visualized using Non-metric Multidimensional Scaling (NMDS) plot with Bray–Curtis dissimilarity distances was also significant in ileal (R^2^ = 0.59; *P* = 0.001) and colonic (R^2^ = 0.41; *P* = 0.003) fecal samples (Fig. 6C,D). Next, DESeq2 analysis was performed to assess differential expression on genus level. Major changes could be observed throughout all groups, yet as expected from earlier results, differences were most pronounced in 21 days treated groups (Fig. 6E–H). We conclude that due to its effect on epithelial proliferation and differentiation, α_2_-AR stimulation also profoundly affects intestinal microbial composition.

### Sympathetic denervation worsens anastomotic wound healing

**Figure 7:**
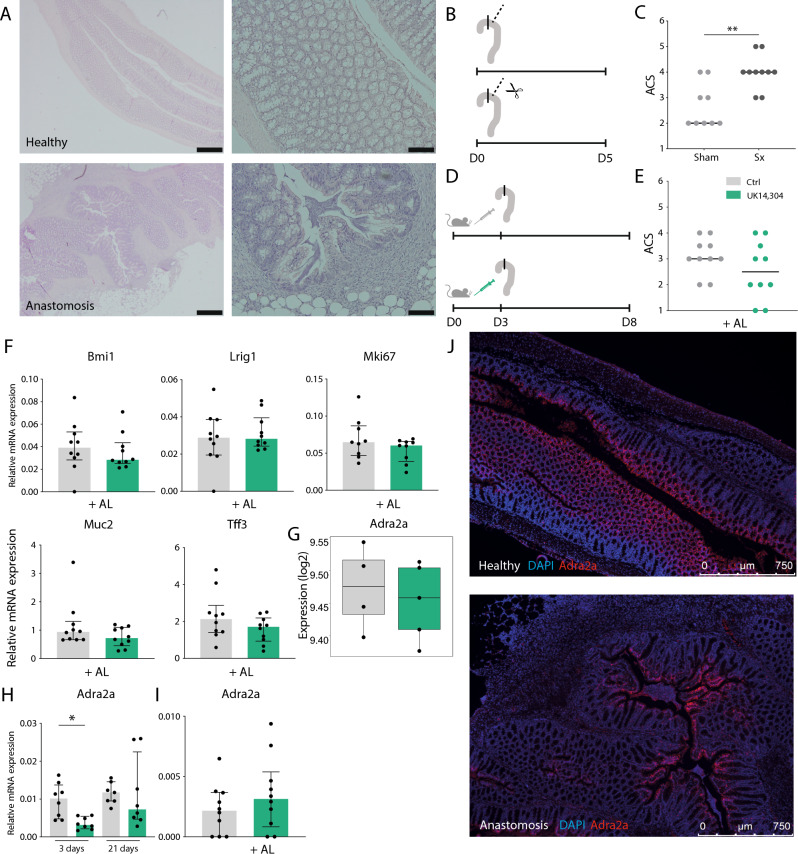
Impact of denervation of cell proliferation. (**A**) Representative pictures H&E stains of healthy colonic tissue (upper panel), and colonic tissue with an anastomosis (lower panel). Magnifications: 20x and 40x. (**B**,**C**). Mice were subjected to surgery in which the mesenteric nerve bundle was cut and an anastomosis was created. Sham group underwent anastomotic surgery without cutting the mesenteric nerve bundle. Mice were euthanized after 5 days (Sham *n* = 9; Sx *n* = 10). (**B**) Experimental set-up. (**C**) Visualization of anastomotic complication score (ACS). Data are shown as individual data points with median. *P* = 0.004. (**D**–**F**). Mice were treated with UK 14,304 (green) or vehicle control (grey) via oral gavage for 3 days after which they were subjected to surgery for creation of an anastomosis. Mice were euthanized after 5 days (*n* = 10). (**D**) Experimental set-up. (**E**) Visualization of anastomotic complication score (ACS). Data are shown as individual data points with median. (**F**) Quantitative RT-PCR (qPCR) analysis of epithelial cell markers *Bmi*, *Lrig1*, *Muc2*, *Tff3*, and proliferation marker *Mki67*. Data are shown as individual data points with median and interquartile range. (**G**) Single gene expression analysis of *Adra2a* in UK 14,304 versus vehicle control treated organoids assessed by bulk RNA-sequencing (experiment Fig. 2A) (*n* = 5). Data are shown in a boxplot. (**H**) qPCR analysis of expression of *Adra2a* in mice treated with UK 14,304 or vehicle control via oral gavage for 3 or 21 days (experiment Fig. 4A) (*n* = 8). Data are shown as individual data points with median and interquartile range. Vehicle control versus UK 14,304 3 days *P* = 0.020. (**I**) qPCR analysis of expression of *Adra2a* in mice treated with UK 14,304 or vehicle control via oral gavage for 3 days after which they underwent anastomotic surgery (experiment Fig. 7D) (*n* = 10). Data are shown as individual data points with median and interquartile range. (**J**) In situ hybridization (RNAscope) for *Adra2a* mRNA in colonic tissue of mice subjected to anastomotic surgery in combination with immunofluorescent stainings with DAPI for nuclei (blue). Single dots (red) represent expression of a single *Adra2a* mRNA copy. Magnification 10x. Statistical significance is indicated as follows: *P < 0.05, **P < 0.01.

Because we show that AR stimulation leads to increased intestinal proliferation and mucus production, we aimed to investigate if sympathetic stimulation would lead to augmented wound healing. First, we showed that α_2_-AR stimulation might benefit wound healing by use of human fetal organoids that were cultured in monolayer by using the ECIS epithelial wounding platform (Supplementary Fig. 5). To explore this further in an in vivo setting, we made use of an anastomotic wound-healing mouse model, in which a vessel-sparing anastomosis without bowel resection is created in the proximal colon. A validated anastomotic complication score^45^ was used as an outcome measure. This score ranges from 1 (excellent healing) to 6 (poor healing) and is determined based on presence of adhesions or other abnormalities, anastomotic defects, signs of contamination, and signs of clear anastomotic complication (e.g. spread of pus, obstruction at anastomosis, peritonitis). Figure 7A shows representative pictures of H&E stains of healthy and anastomotic tissue. We first aimed to study the effect of inadequate sympathetic activity on wound healing by subjecting mice to the combined creation of anastomosis and mesenteric nerve plexus denervation (or sham) (Fig. 7B). In accordance with our expectations, lack of sympathetic innervation led to worsening of wound healing as was shown by increased ACS when compared to sham animals (*P* = 0.004) (Fig. 7C).

We next investigated the molecular effect of AR stimulation in our anastomotic healing model. Mice undergoing anastomotic surgery were pre-treated for 3 days with UK 14,304 or vehicle control via oral gavage (Fig. 7D). Although an improved healing was seen along with a reduction of ACS, results were not significant in our tested group (Fig. 7E). mRNA expression levels of epithelial cell markers and proliferation marker *Mki67* were assessed by qPCR analysis (Fig. 7F). Results did not show an increase of the stem cell markers or proliferation marker *Mki67*, or a decrease of the differentiation markers that were observed earlier compared to the control group.

We argued that a potential cause could be a change in α_2A_-AR availability. Therefore, we studied *Adra2a* expression levels in experimental groups. In bulk RNA-seq data (epithelial cells) *Adra2a* expression was unaltered upon UK 14,304 treatment (Fig. 7G). In vivo data of healthy mice receiving UK 14,304 showed *Adra2a* downregulation (*P* = 0.020) (Fig. 7H), whereas this effect was reversed in mice undergoing anastomotic surgery after oral gavage with UK 14,304 (Fig. 7I). Figure 7J shows representative pictures of *Adra2a* expression as assessed by RNAscope in intestinal anastomotic tissue of mice that underwent anastomotic surgery (Supplementary Fig. 2E) demonstrating an elevated expression in tissue surrounding the anastomotic site.

## Discussion

Epithelial stem cell function is critical for intestinal wound healing. Here, we report that sympathetic activity through the α_2A_-AR supports this function, and that α_2A_-AR is the most abundant AR present in the intestinal epithelium. Our data demonstrate that α_2A_-AR mediates sympathetic control of intestinal epithelial cell proliferation and cell composition. Specific α_2A_-AR stimulation results in enhanced epithelial cell proliferation and stem cell function. Also, it leads to profound changes in the microbial composition in the intestinal lumen, likely through changes in the epithelial secretion of antimicrobial peptides. Additionally, we show that the increased cell proliferation resulting from α_2A_-AR stimulation is the consequence of altered MAPK signaling. This study confirms previous reports^6,9^ and strengthens outcomes with in vivo data. Also, this supports our earlier findings that the sympathetic nervous system plays a crucial role in modulating mucosal epithelial cell function^44^.

Our studies demonstrate profound enhancement of mucus production upon α_2A_-AR stimulation compared to controls. The overall expanded mucin load led to the increased abundance of mucin degraders like *Bifidobacterium*, *Ruminococcus*, and *Akkermansia* as expected. Of interest, it has recently been demonstrated that *Akkermansia* can promote intestinal stem cell-mediated epithelial development and contribute to intestinal homeostasis maintenance^46^. In fact, all mucus-degrading bacteria were suggested to have this effect. Spore-forming Gram-positive bacteria like *Clostridium* species (showing an increased abundance in our experiments) similarly promote intestinal epithelial cell turnover^47^. This indicates that epithelium and microbiome converge in promoting cell proliferation. In light of the major microbiome changes as result of the α_2A_-AR stimulation, it should be noted that microbial composition can be altered by intestinal motility^48^. Since UK 14,304 is known to influence peristalsis leading to intestinal flow and mixing^49^, we cannot rule out that experimental results on microbiome composition may be partly explained by altered motility.

Denervation of the sympathetic mesenteric nerve plexus supplying intestinal tissue negatively affected the anastomotic healing process, and specific sympathetic α_2A_-AR stimulation might reverse this. We reason that several factors can explain this. Of course, wound healing is more complex than epithelial proliferation and regeneration and has to be studied in context. Processes could be affected by the preceding hemostatic and inflammatory states of the tissue, although earlier research demonstrated that α_2A_-AR expression is not affected by inflammation^50^. In our studies, the anastomotic healing mouse model was used. This is an invasive model involving all layers of the intestine. In further studies, a model particularly focusing on the epithelium could be of use. As demonstrated, the ECIS wound healing assay for organoid monolayers has great potential for such studies. Since it was shown that in these models variation between samples and wounding is extensive^51^ large group sizes are required in further studies. In the anastomotic healing mouse model effects were studied in colonic tissue, whereas other experiments were performed using small intestinal tissue. *Adra2a* mRNA expression is found to be upregulated upon stimulation in ileum, whereas downregulation is observed in colonic samples suggesting that the proliferative effect of α_2A_-AR stimulation in colon could be to a lesser extent compared to ileum. Alternating levels of α_2A_-AR expression along the proximal–distal intestinal tract in humans were demonstrated before^7^. This could be the case in mice as well and offers an explanation for tissue location-specific alterations. Differences in results could also be explicated by region-specific responses of sympathetic input^52^. Furthermore, differences in AR availability, and neurotransmitter concentration (impacting receptor downregulation or desensitization, as was discussed elsewhere^14^) could modify effects. This is relevant in light of the differences in expression levels of α_2A_-AR throughout our experiments. Optimally, knockout models should be considered to confirm the specific role of a receptor. However, for α_2A_-AR, these are less appropriate since many physiological compensatory mechanisms in AR knockout models have been documented^53–55^. Further studies are needed, preferably including scRNA-seq experiments to elucidate epithelial functions elicited through adrenergic activation in distinct epithelial subsets.

Sympathetic innervation is decreased during intestinal inflammation seen in IBD^13^, supported by our earlier observation that AR blocker usage is positively associated with IBD relapse episodes^56^. Our study is in line with such data since we demonstrate that sympathetic activity elicits promotion of mucosal healing at the level of epithelia. Currently available treatments for IBD mostly act by inhibiting inflammatory processes through the blockage of specific inflammatory molecules. Paradoxically, many of these hinder the process of mucosal healing. The effect of TNF-α for instance, promoting intestinal mucosal repair through Platelet Activating Factor receptor signaling, is blocked by common monoclonal antibodies like infliximab and adalimumab^57^. Ustekinumab targeting the Th17 pathway inhibits the production of IL-22, a modulator of epithelial repair^58^ and a factor involved in the stimulation of Paneth cell formation and reducing stem cell activity^59^.

With this study, we shed light on the mechanism through which sympathetic neural activity can control epithelial function. Together, we establish that epithelial α_2A_-AR is a molecular relay of sympathetic innervation to affect stem cell activity and wound healing. The potential of bioelectronic neuromodulation in ameliorating inflammation in IBD has been studied in basic^1,60–65^ and clinical^66–70^ context. This current study may reveal potential clinical application of improving epithelial barrier and mucosal healing by modulating sympathetic activity through electrical stimulation or pharmacological agonists. As such, bioelectronic devices designed to activate sympathetic nerves promote the release of norepinephrine and seem challenging to tailor for specific ARs. Yet, our data lead to a better understanding of the molecular keys of sympathetic control over epithelial functions and host defense.

### Supplementary Information

Supplementary Information.

## Data Availability

All RNA sequencing data are deposited in the Gene Expression Omnibus (GEO; GSE230088).

## Abbreviations

AR: Adrenergic receptor
IBD: Inflammatory bowel disease
