# Supplementary material for: Sympathetic activity regulates epithelial proliferation and wound healing via adrenergic receptor α2A
Source: Sci Rep. 2023 Oct 20;13:17990. doi: 10.1038/s41598-023-45160-w (PMC10589335; doi:10.1038/s41598-023-45160-w)
Supplement: Supplementary file 1 — Supplementary Information. [file 41598_2023_45160_MOESM1_ESM.pdf]

Sympathetic activity regulates epithelial proliferation and wound healing via adrenergic receptor α_{2A}

Anne S. ten Hove[1,&,*], Shilpashree Mallesh[2,&], Konstantina Zafeiropoulou[1], Janna W. M. de Kleer[1], Patricia H.P. van Hamersveld[1], Olaf Welting[1], Theo Hakvoort[1], Sven Wehner[2], Jurgen Seppen[1], Wouter J. de Jonge[1,2]

1. Tytgat Institute for Liver and Intestinal Research, Amsterdam UMC, University of Amsterdam, Gastroenterology and Hepatology, Amsterdam Gastroenterology Endocrinology Metabolism, Amsterdam, the Netherlands
2. Department of General, Visceral-, Thoracic and Vascular Surgery, University Hospital Bonn, Germany.

&. Joint first authorship.

*. Corresponding author: a.s.tenhove@amsterdamumc.nl, Tel. +3120-5668163, Meibergdreef 69-71, 1105 BK Amsterdam, The Netherlands.

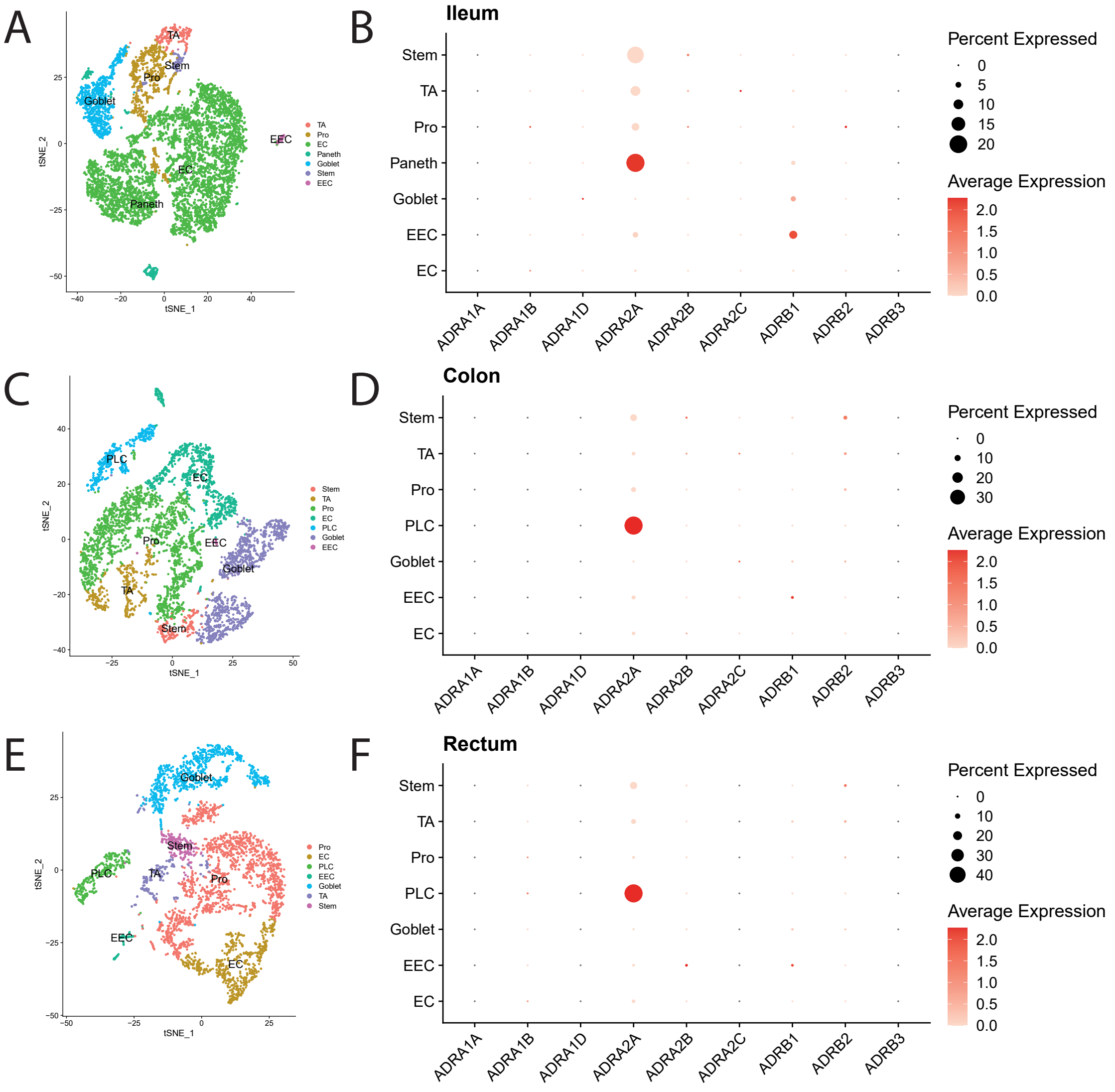

Supplementary Figure 1. Intestinal epithelial cell expression of adrenergic receptors in human tissues. **A-F.** Single-cell RNA-sequencing of human intestinal epithelial cells. Data were obtained from GSE125970. **A, C, E.** t-distributed stochastic neighbor embedding (t-SNE) visualization of the unsupervised clustering analysis of all cells. **B, D, F.** Dot Plot visualization of the expression of adrenergic receptors in cell type classes. Ileum is shown in A-B, colon in C-D, and rectum in E-F.

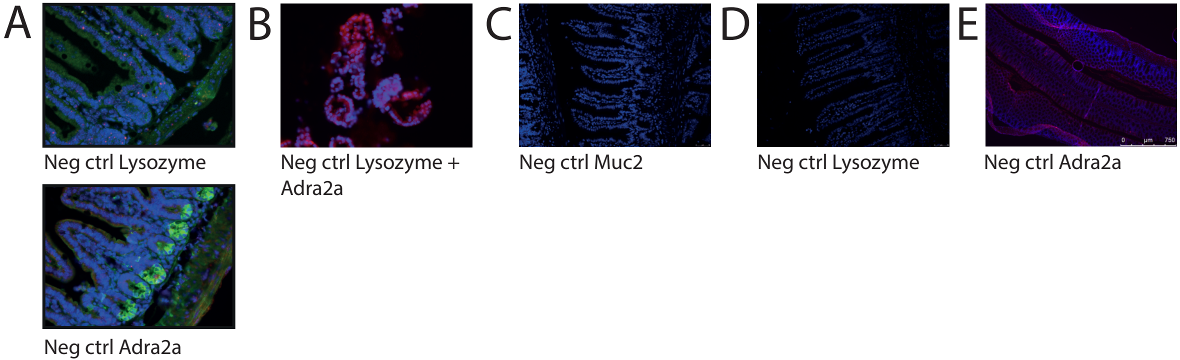

Supplementary Figure 2. Negative controls of in situ hybridization and immunofluorescent pictures. **A-E.** All negative controls for in situ hybridization (RNAscope) for Adra2a mRNA in mouse small intestine in combination with immunofluorescent stainings with DAPI for nuclei (blue) and lysozyme (green). Single dots (red) represent expression of a single Adra2a mRNA copy. Magnification: 20x (D) or 40x (E).

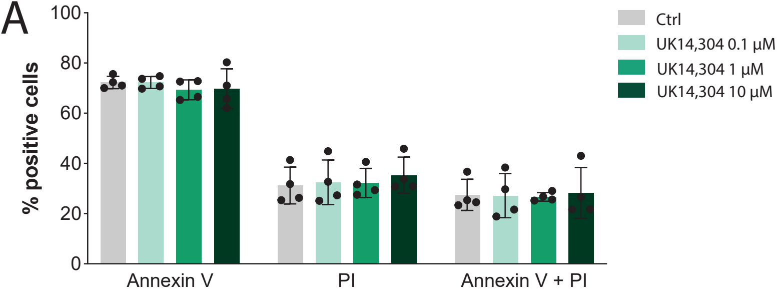

Supplementary Figure 3. Apoptosis assay of mouse small intestinal organoids upon α2A-AR stimulation. A. In vitro apoptosis assay of mouse small intestinal organoids that were stimulated with UK 14,304 in concentrations 0.1 µM, 1 µ M, and 10 µM, or vehicle control (n=5). Apoptosis was measured using FACS analysis with markers for Annexin V and propidium iodide (PI). Data are shown as individual data points with mean and standard deviation.

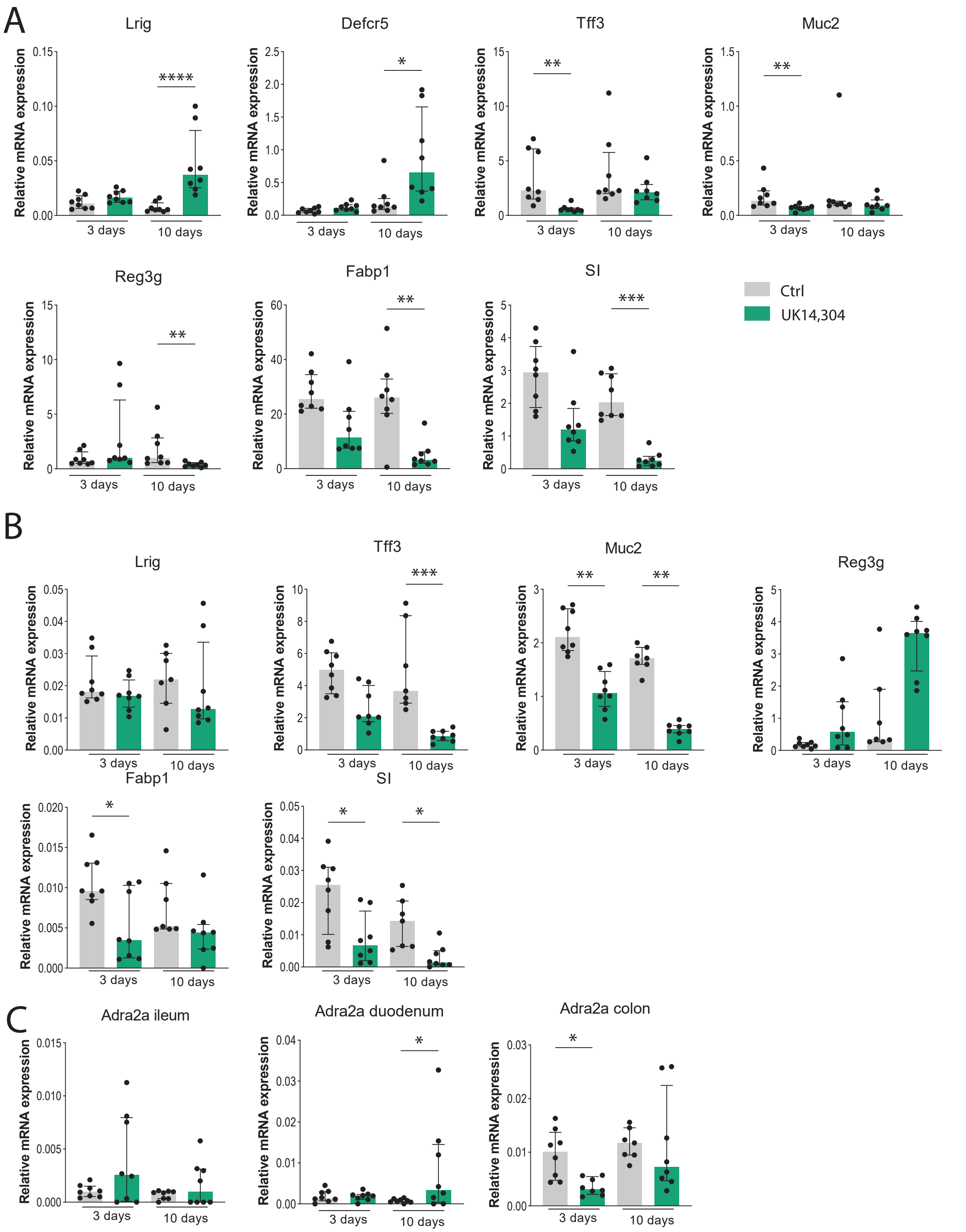

Supplementary Figure 4. Assessment of α2A-AR stimulation on stem cell function in vivo. **A.** Quantitative RT-PCR (qPCR) analysis of expression of epithelial stem cell markers, differentiation markers *Lrig1* (vehicle control vs UK 14,304 21 days P<0.0001), Defcr5 (vehicle control vs UK 14,304 21 days P<0.041), *Tff3* (vehicle control vs UK 14,304 3 days P=0.001), *Muc2* (vehicle control vs UK 14,304 3 days P=0.004), *Reg3g* (vehicle control vs UK 14,304 21 days P=0.005), *Fabp1* (vehicle control vs UK 14,304 21 days P=0.003), and SI (vehicle control vs UK 14,304 21 days P=0.0008) in mouse duodenum. Data are shown as individual data points with median and interquartile range. **B.** Quantitative RT-PCR (qPCR) analysis of expression of epithelial stem cell markers, differentiation markers *Lrig1, Tff3* (vehicle control vs UK 14,304 21 days P=0.0008), *Muc2* (vehicle control vs UK 14,304 3 days P=0.005 and 21 days P=0.001), Reg3g, Fabp1 (vehicle control vs UK 14,304 21 days P=0.025), *SI* (vehicle control vs UK 14,304 3 days P=0.045 and 21 days P=0.025) in mouse colon. Data are shown as individual data points with median and interquartile range. **C.** Quantitative RT-PCR (qPCR) analysis of expression of *Adra2a* in mouse ileum, duodenum (vehicle control vs UK 14,304 21 days P=0.028), and colon (vehicle control vs UK 14,304 3 days P=0.020). Data are shown as individual data points with median and interquartile range.
Statistical significance is indicated as follows: *P<0.05, **P<0.01, ***P<0.001, ****P<0.0001.

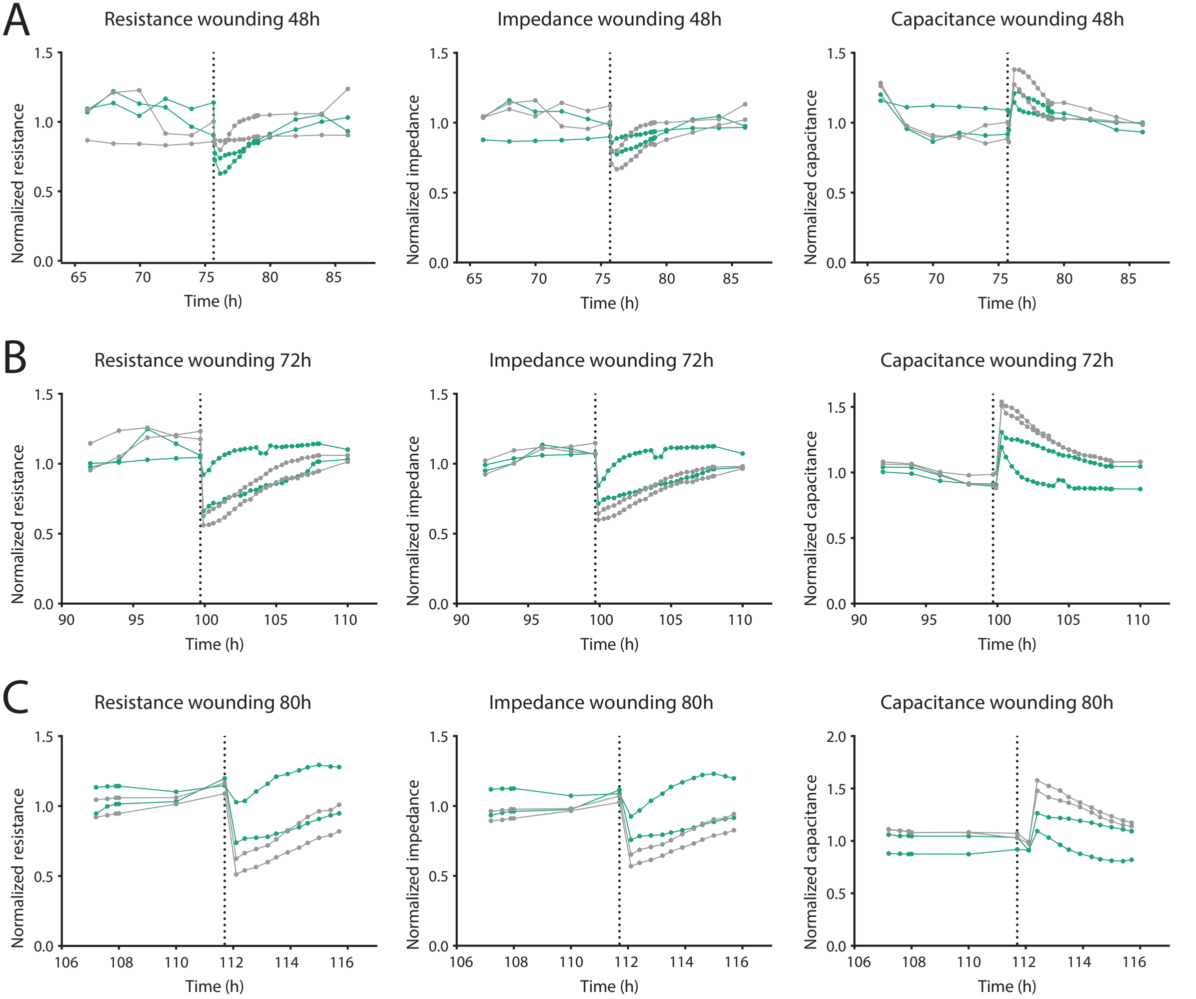

Supplementary Figure 5. Analysis of real-time barrier integrity of human fetal organoid monolayer upon α2A-AR stimulation following wounding on 8W10E array. Impedance gives insight into aspects of cellular behaviors and movements, resistance provides information on barrier integrity, and capacitance is indicative for cell migration. Organoid monolayers were treated with UK 14,304 (green) or vehicle control (grey). **A.** Time-course of normalized resistance, impedance, and capacitance measurements following electrical wounding 48 hours after treatment with UK 14,304 (green) or vehicle controls (grey) (n=2). **B.** Time-course of normalized resistance, impedance, and capacitance measurements following electrical wounding 72 hours after treatment with UK 14,304 (green) or vehicle controls (grey) (n=2). **C.** Time-course of normalized resistance, impedance, and capacitance measurements following electrical wounding 80 hours after treatment with UK 14,304 (green) or vehicle controls (grey) (n=2). Dashed lines mark time of wounding.

Supplementary Table 1. Primers

**Target (all mouse)**	**Forward sequence**	**Reverse sequence**
Adra1a	TTTCTTGAACTCCTGGCTGG	CTGCCATTCTTCCTCGTGAT
Adra1b	AGCTGTTGAAGTAGCCCAGC	AACCTTGGGCATTGTAGTCG
Adra1d	GATGGTTTCAGCTGAGGGAA	TCCGTAAGGCTGCTCAAGTT
Adra2a	CGGAGTTGGAAATCTCTTCG	GGCCTGATGATTTGGAAAGA
Adra2b	AATCATGATGAGGCAGGGAG	GCAGCCGTCATTTCTCTACC
Adra2c	GCTGGTTCCCCTTCTTCTTC	CTTGAAAGAGCGCCTGAAGT
Adrb1	CACACAGGGTCTCAATGCTG	GATCTGGTCATGGGATTGCT
Adrb2	CAGGAACTGCTGTGTGAGGA	CCTTGGGAGTCAACGCTAAG
Adrb3	TAGTTCCCAGCGGAGTTTTC	AGCCATCAAACCTGTTGAGC
Olfm4	GCCACTTTCCAATTTCAC	GAGCCTCTTCTCATACAC
Lyz1	AAGAATGCCTGTGGGATCAA	CGGTTTTGACATTGTGTTCG
Muc2	TGAAGACCGAGATTGTGCCC	AGATGACGTTGAGCTGGGTG
Bmi1	CCAATGAAGACCGAGGAGAA	TTTCCGATCCAATCTGCTCT
Tff3	TTGCTGGGTCCTCTGGGATAG	TACACTGCTCCGATGTGACAG
Mki67	ACCGTGGAGTAGTTTATCTGGG	TGTTTCCAGTCCGCTTACTTCT
Lgr5	TGTGTCAAAGCATTTCCAGC	CAGCGTCTTCACCTCCTACC
Vil1	CTCAAGACTCCGTCCTGCTG	CCACTTGTTTCTCCGTCCGA
SI	CCAGACACCCCTACAAGCTC	GTGTTCCGCTTCCCCAAAAC
Fabp1	CGTTGCCACCATGAACTTCT	TGAACTCATTGCGGACCACT
Alpi	AGGACATCGCCACTCAACTC	GGTTCCAGACTGGTTACTGTCA
Chga	CCAAGGTGATGAAGTGCGTC	GGTGTCGCAGGATAGAGAGGA
Reg3g	TCCACCTCTGTTGGGTTCAT	AAGCTTCCTTCCTGTCCTCC
Defcr5	AGGCTGATCCTATCCACAAAACAG	TGAAGAGCAGACCCTTCTTGGC

Supplementary Table 2. Alpha diversity measures (used for Figure 6)

	Measure	From	To	y	P
**Ileum**	Richness	21_V	21_UK	677.7	0.014
	Richness	3d_V	3d_UK	699.4	0.087
	Shannon	21_V	21_UK	3.844776	0.03
	Shannon	3d_V	3d_UK	3.945245	0.046
**Colon**	Richness	21_UK	21_V	1296.2	2.50E-07
	Richness	3d_V	3d_UK	1326.4	0.022
	Shannon	21_UK	21_V	5.183186	1.30E-08
	Shannon	3d_V	3d_UK	5.267265	0.0039

Supplementary Table 3. Beta diversity measures (used for Figure 6)

		Df	SumsOfSqs	MeanSqs	F.Model	R2	Pr(>F)
**Ileum**	Groups	3	0.080036	0.0266785	6.1612	0.40638	0.003 **
	Residuals	27	0.116912	0.0043301		0.59362	
	Total	30	0.196948			1	
**Colon**	Groups	3	0.5089	0.169632	13.24	0.58654	0.001 ***
	Residuals	28	0.35873	0.012812		0.41346	
	Total	31	0.86763			1	

Original western blots (Figure 3)

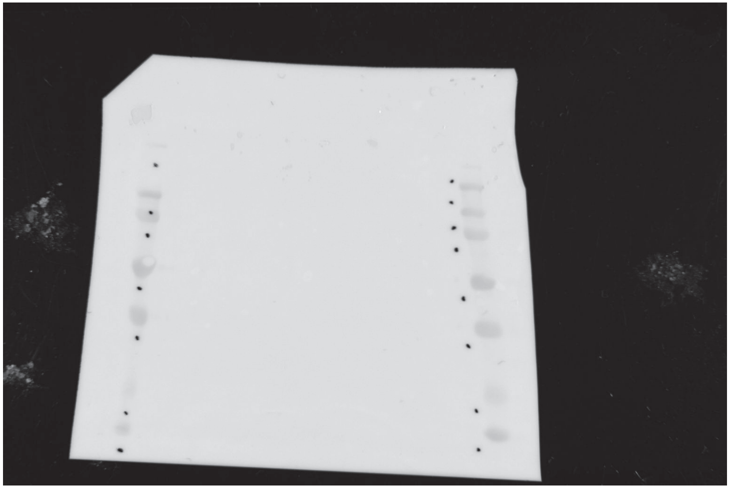

p-ERK ladder

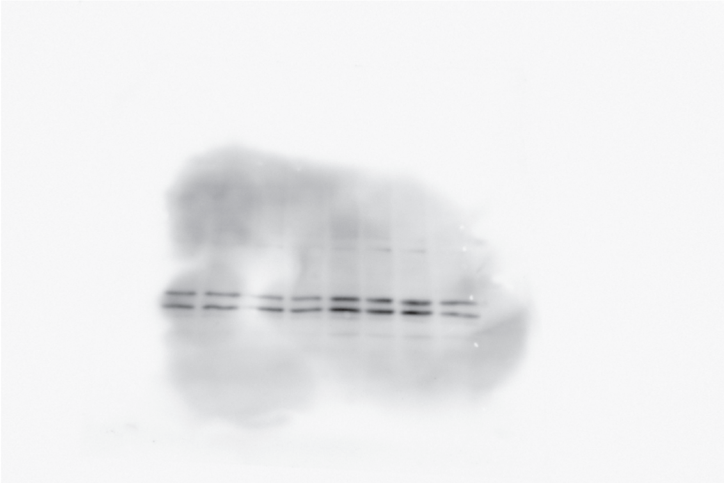

p-ERK

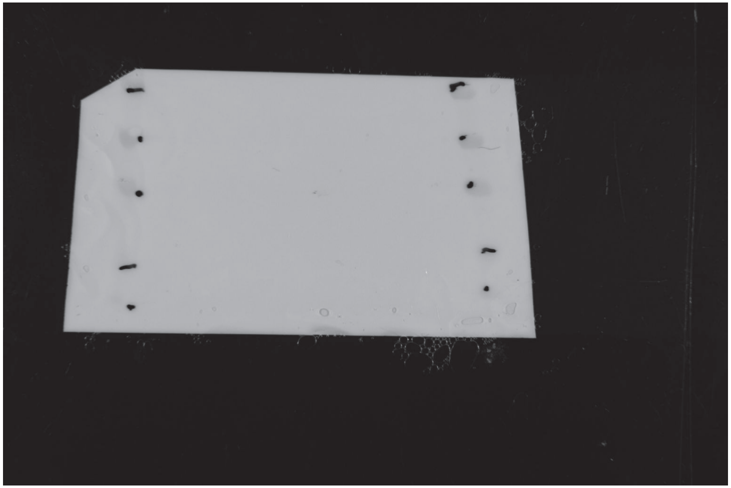

ERK ladder

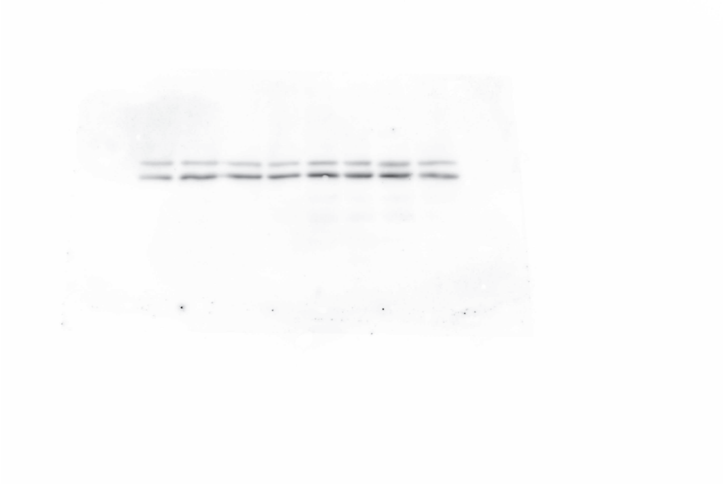

ERK
